# The Incidence and Associated Risk Factors of Contrast-Induced Nephropathy after Contrast-Enhanced Computed Tomography in the Emergency Setting: A Systematic Review

**DOI:** 10.3390/life12060826

**Published:** 2022-06-01

**Authors:** Mei-Yi Ong, Justin Jie-Hui Koh, Suchart Kothan, Christopher Lai

**Affiliations:** 1 Health and Social Science Cluster, Singapore Institute of Technology, 10 Dover Drive, Singapore 138683, Singapore; 1800433@sit.singaporetech.edu.sg (M.-Y.O.); 1800135@sit.singaporetech.edu.sg (J.J.-H.K.); 2 Center of Radiation Research and Medical Imaging, Department of Radiologic Technology, Faculty of Associated Medical Sciences, Chiang Mai University, Chiang Mai 50200, Thailand; suchart.kothan@cmu.ac.th

**Keywords:** contrast-induced nephropathy, contrast media, contrast-enhanced computed tomography, emergency department

## Abstract

Iodinated contrast media (ICM) during contrast-enhanced computed tomography (CECT) in the emergency department (ED) is essential to diagnose acute conditions, despite risks of contrast-induced nephropathy (CIN) development and its associated complications. This systematic review aims to evaluate the incidence of CIN and CIN-induced complications, and to explore the relevance of classical risk factors for CIN among ED patients receiving ICM. PubMed, Cochrane, and Web of Science were used on 30 August 2021 to search for peer-reviewed English articles reporting on CIN incidence among ED patients aged ≥18 years who underwent an intravenous CECT. The inclusion criteria included studies that were in English, peer-reviewed, and involved ED patients aged ≥18 years who underwent single intravenous CECT. Studies on intra-arterial procedures and preventive strategies, meta-analyses, clinical guidelines, review articles, and case reports were excluded. The JBI critical appraisal checklist was applied to assess the risk of bias. In total, 18 studies were included wherein 15 were retrospective studies while three were prospective studies. We found a relatively higher CIN incidence in the ED, with variations owing to the CIN definitions. Several classical risk factors including acute hypotension remain linked to CIN onset in ED settings unlike factors such as age and diabetes. While risk of adverse renal events due to CIN is low, there is higher risk of CIN-induced mortality in the ED. Therefore, with the higher incidence of CIN and CIN-induced mortality rates in the ED, ICM administration during CECT in the ED should still be clinically justified after assessing both benefits and risks.

## 1. Introduction

Computed tomography (CT) utilization in the emergency department (ED) has been increasing globally over the years [1,2]. Iodinated contrast media (ICM) administration during contrast-enhanced CT (CECT) in the ED is crucial for the accurate diagnosis of acute conditions such as aortic dissection and pulmonary embolism [3,4]. As ICM usage has been linked to adverse effects such as contrast-induced nephropathy (CIN) or contrast-associated acute kidney injury (CA-AKI), screening for patients with increased risk of CIN development, which involves serum creatinine (sCr) assessment and identification of classical risk factors such as diabetes, prior to ICM administration is recommended in most clinical situations. Therefore, for patients at higher risk of CIN, CECT is usually postponed or substituted with non-CECT examinations or alternative imaging modalities such as ultrasound [5]. However, in emergencies, clinicians may omit certain routine screening processes and proceed with CECT if the benefits outweigh its risks [6]. Therefore, this could potentially place ED patients at greater risk for developing CIN.

CIN is characterized by a sudden decline in renal function due to ICM administered intravascularly [7]. Currently, there is no universally accepted CIN definition and hence, varying definitions have been used across multiple studies. Two common definitions used include the Kidney Disease Improving Global Outcomes (KDIGO) and the Acute Kidney Injury Network (AKIN) criteria. The KDIGO and AKIN criteria define CIN as an absolute sCr increase of ≥0.3 mg/dL above baseline (i.e., sCr level before ICM administration) within 48 h, a reduction of urine output to ≤0.5 mL/kg/h for 6 h or more; or a percentage increase in sCr of ≥50% above baseline within 7 days or 48 h, respectively [8,9].

Several reports have studied CIN incidence in the ED. Turedi et al. [10] revealed a CIN incidence as high as 23.7%, despite the use of at least one prophylaxis before and after CECT for all ED patients. Similarly, a high CIN incidence of 14.7% was reported in a South African study targeting trauma patients who underwent CECT [11]. Conversely, Dağar et al. [12] observed a low CIN incidence of 4.9% among adult patients in the ED, while McGillicuddy et al. [13] demonstrated an even lower CIN incidence of 1.9%. Overall, wide variations in CIN incidence after CECT among ED patients were reported in multiple studies, which could be due to several factors such as patients’ underlying conditions and different CIN definitions used. 

Furthermore, CIN has been reported to be associated with greater risk of major adverse events in the ED such as renal failure and death, with a two-fold increase in risk of 1 year adverse events within 1 year among CIN individuals after CECT [14]. Mild increases in sCr are also associated with a higher risk of short-term mortality across different clinical settings and patient types [15].

Currently, there is no systematic review investigating the incidence of CIN in the ED and its associated complications due to CIN onset. Additionally, the topic on whether classical risk factors studied in largely non-ED settings are still valid for ED patients receiving ICM during CECT has not been extensively studied. Therefore, this systematic review aims to (1) evaluate the incidence of CIN and CIN-induced complications among adult patients after receiving ICM during CECT in the ED, and (2) explore the relevance of classical risk factors for CIN in the ED for CECT patients receiving ICM.

## 2. Materials and Methods

### 2.1. Search Strategy

An initial set of search terms was developed using variations of the keywords: “contrast-induced nephropathy”, “contrast-enhanced computed tomography” and “emergency department”. A preliminary search was conducted using PubMed and search terms were subsequently improved on using relevant MeSH descriptors and search operators. The final search was performed on 30 August 2021 using PubMed, Cochrane, and Web of Science databases with no search filters applied. The full search strategy is documented in Table 1.

### 2.2. Eligibility Criteria

The inclusion criteria included studies that were in English, peer-reviewed, and involved ED patients aged ≥18 years who underwent single intravenous CECT. The studies should also have a clear definition of CIN, with CIN incidence reported as an outcome. There were no restrictions to the country and publication date. Studies on intra-arterial procedures and preventive strategies, meta-analyses, clinical guidelines, review articles, and case reports were excluded.

### 2.3. Study Selection

After excluding duplicates, the title and abstract of the studies were independently screened by two reviewers based on the inclusion and exclusion criteria. If the eligibility of articles could not be determined due to inadequate information, the full papers were retrieved and assessed. Subsequently, the Joanna Briggs Institute (JBI) critical appraisal checklists for cohort and case control studies were used by the same two reviewers to review the methodological quality of the selected articles [16]. Throughout the process, any disagreement between the reviewers was discussed until consensus was achieved. The search and study selection were done in accordance with the Preferred Reporting Items for Systematic Reviews and Meta-Analyses (PRISMA) guidelines [17].

### 2.4. Data Collection

Using a self-generated data extraction form, data were extracted from all included studies by one reviewer and subsequently checked by the other reviewer to minimize errors. The data included the author, publication year, study design, country of origin, the type and number of patients, definition of nephropathy, type and volume of ICM, CT scan region, CIN incidence after single CECT, dialysis, and mortality statistics.

## 3. Results

### 3.1. Study Identification

**Figure 1:**
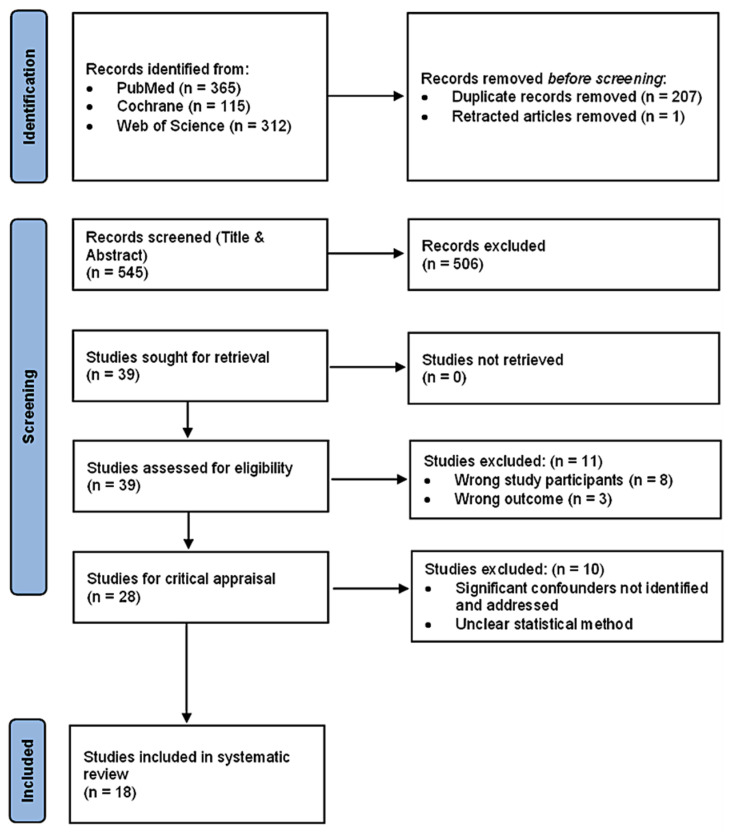
PRISMA Flow Diagram.

A total of 792 articles were identified using PubMed (n = 365), Cochrane (n = 115) and Web of Science (n = 312). After eliminating 207 duplicates and one retracted article, 545 articles remained for screening by title and abstracts according to the eligibility criteria. A total of 506 articles were subsequently excluded and the full papers of the remaining 39 articles were retrieved and reviewed. Of these, 11 articles were removed due to wrong study participants (n = 8) and wrong outcome reported (n = 3). The JBI critical appraisal checklist was applied to the remaining 28 articles and 10 articles were further excluded as a result of one or both reasons. These reasons are the lack of identifying and addressing significant confounders, and unclear statistical methods. Finally, a total of 18 articles were included (Figure 1).

### 3.2. Study Characteristics

The study characteristics are summarized in Table 2. Of the 18 included studies, 15 were retrospective studies while three were prospective studies. While the primary population targeted was ED adult patients, some studies focused on specific groups including only elderly patients, those with stage 3 to 5 chronic kidney disease (CKD), and patients with sepsis, acute ischemic stroke (AIS) or active cancer (Table 2). 

It should be noted that Hong et al. [18] had active cancer patients who underwent two or more CECT scans. However, this article was still included as we were able to isolate CIN cases with single CECT and calculate the incidence based on the data given. 

The incidence of CIN varies greatly, ranging from 1.9% to 36.9%, with sample size from 105 to 7201 (Table 2). Due to the wide variation of CIN incidence across different studies, there is no standardized reference value to categorize the incidence of CIN as high or low. Hence, we decided to use the pooled CIN incidence of 4.96% from the meta-analysis by Moos et al. [19], which focused mainly on non-ED patients receiving CECT, as the cut-off value to classify as high or low CIN incidence. A total of 14 different definitions were used, with the most common one being an absolute increase of ≥0.5 mg/dL or ≥25% increase over baseline sCr, occurring within 48 to 72 h. When the definitions were further segregated, there were eight different sCr measurements, defined within five varied time intervals. Dialysis and mortality among CIN patients were also discussed in fourteen studies and eight studies, respectively.

**Table 2:** Study Characteristics.

Author and Year	Study Design	Country	CECT Patients (n)	Patient Type	Definition of Nephropathy		Contrast Media		CT Scan Coverage	Incidence of CIN (High/Low Incidence *)	Dialysis	Mortality
					Measurement of sCr	Post-CECT sCr Collection Time	Type	Volume (mL)				
Kene et al., 2021 [20]	Retrospective Cohort	USA	5589	Adult patients with CKD stage 3 to 5	Absolute sCr increase of ≥0.3 mg/dL or ≥1.5-fold increase over baseline sCr	24 to 72 h	LOCM	75–150	Head, neck, chest, abdomen or pelvis	13.2% (High)	0.7% (39 patients)	7.1% (397 deaths)
Brito et al., 2020 [21]	Retrospective Cohort	Portugal	161	Adult patients with acute ischemic stroke	Absolute sCr increase of ≥0.3 mg/dL or ≥1.5-fold increase over baseline sCr	Within 72 h	LOCM	90	Brain	6.2% (High)	0.6% (1 patient)	NM
Akman and Bakirdogen, 2020 [22]	Retrospective Cohort	Turkey	122	Adult patients	Absolute increase of ≥0.5 mg/dL or ≥25% Increase over baseline sCr	Within 72 h	NM	NM	All regions	36.9% (High)	NM	NM
Dağar et al., 2020 [12]	Retrospective Cohort	Turkey	631	Adult Patients	Absolute increase of ≥0.5 mg/dL or ≥25% increase over baseline sCr	48 to 72 h	LOCM	100	Chest, abdomen or pelvis	4.9% (Low)	0.2% (1 patient)	0%
Hinson et al., 2019 [23]	Retrospective Cohort	USA	1464	Adult patients with sepsis	Absolute sCr increase of ≥0.3 mg/dL or ≥1.5-fold increase over baseline sCr	48 to 72 h	LOCM/IOCM	80–120	All regions	7.2% (High)	NM	NM
Cho et al., 2019 [24]	Retrospective Cohort	South Korea	632	Adult Patients	Increase in sCr ≥ 0.3 mg/dL or ≥1.5 to 1.9-fold increase over baseline sCr	48 to 72 h	LOCM	60	Chest	6.49% (High)	0.79% (5 patients)	NM
Hsu et al., 2019 [25]	Retrospective Cohort	Taiwan	105	Adult patients with sepsis	Absolute increase of 0.5 mg/dL or >50% increase over baseline sCr	48 to 72 h	LOCM	Up to 120	All regions	12.4% (High)	10.5% (11 patients)	25.7% (27 deaths)
Puchol et al., 2019 [26]	Retrospective Cohort	Spain	6642	Adult patients	(1) Absolute increase of ≥0.3 mg/dL or 1.3 times greater than baseline sCr (2) Absolute increase of ≥0.5 mg/dL or ≥25% Increase over baseline sCr	24 to 72 h	LOCM	50–200	All regions	(1) 7.15% (High) (2) 7.72% (High)	NM	NM
Hinson et al., 2017 [27]	Retrospective Cohort	USA	7201	Adult patients	(1) Absolute increase of ≥0.5 mg/dL or ≥25% increase over baseline sCr (2) Increase in sCr ≥ 0.3 mg/dL or ≥1.5 to 1.9-fold increase over baseline sCr	48 to 72 h	LOCM/IOCM	80–120	All regions	(1) 6.8% (High) (2) 10.6% (High)	0.4% (27 patients)	NM
Hong et al., 2016 [18]	Retrospective Cohort	South Korea	820	Adult patients with active cancer	Absolute increase of ≥0.5 mg/dL or ≥25% increase over baseline sCr	48 to 72 h	LOCM	80–150	All regions	7.5% **(High)	0.1% (1 patient)	0.8% (7 deaths: 1 renal failure related)
Huang et al., 2013 [28]	Retrospective Cohort	Northern Taiwan	594	Adult patients aged 65 and above	Increase in sCr ≥ 0.5 mg/dL	48 to 72 h	NM	92.2–105	Chest or abdomen	8.6% (High)	0.5% (3 patients)	13.1% (78 deaths)
Traub et al., 2013 [29]	Retrospective Case-control	USA	5006	Adult Patients	Absolute increase of ≥0.5 mg/dL or ≥25% increase over baseline sCr	48 to 96 h	NM	NM	All regions	7% (High)	NM	NM
Mitchell et al., 2012 [30]	Prospective Cohort	USA	174	Adult Patients	Absolute increase of ≥0.5 mg/dL or ≥25% increase over baseline sCr	2 to 7 days	LOCM	NM	Chest	14% (High)	1.7% (3 Patients)	3% (6 deaths: 2 renal failure related)
Sinert et al., 2012 [31]	Retrospective Cohort Study	USA	773	Adult Patients	Absolute increase of ≥0.5 mg/dL or ≥25% increase over baseline sCr	48 to 72 h	LOCM/IOCM	100–110	Chest, abdomen or pelvis	5.69% (High)	0%	0.5% (4 deaths)
McGillicuddy et al., 2010 [13]	Retrospective Cohort	USA	822	Adult patients aged 55 or older	Absolute increase of ≥0.5 mg/dL or ≥25% increase over baseline sCr	Within 72 h	LOCM	100	All regions	1.9% (Low)	0.3% (2 patients)	NM
Mitchell et al., 2010 [32]	Prospective Cohort	USA	633	Adult Patients	(1) Absolute increase of ≥0.5 mg/dL or ≥25% increase over baseline sCr (2) An absolute increase in sCr of ≥0.3 mg/dL	2 to 7 days	LOCM	NM	All regions	(1) 11% (High) (2) 6% (High)	0.8% (5 patients)	0.9% (6 deaths: 4 renal failure related)
Hopyan et al., 2008 [33]	Retrospective Cohort	USA	198	Adult patients with acute ischemic stroke	≥25% increase in baseline sCr	Within 72 h	LOCM/IOCM	Up to 90	Brain	2.9% (Low)	0%	NM
Mitchell and Kline, 2007 [34]	Prospective Cohort	USA	1224	Adult patients	Absolute increase of ≥0.5 mg/dL or ≥25% increase over baseline sCr	2 to 7 days	LOCM	120	Chest	12% (High)	0%	NM

NM: Not mentioned; LOCM: Low-osmolar contrast media; IOCM: Iso-osmolar contrast media. *: With reference to a systematic review by Moos et al. [19], the reported pooled incidence of 4.96% is used as the cutoff value to categorize the CIN incidence as either high or low incidence. **: Incidence of CIN calculated based on patients with only 1 CECT.

### 3.3. Outcomes

Five main themes were synthesized from the studies (Table A1).

#### 3.3.1. Overall CIN Incidence

The incidence of CIN in the ED ranges widely across the 18 articles. Based on the cut-off value of 4.96%, a majority of the studies (n = 15) reported high incidence of CIN ranging from 5.69% to 36.9%. The remaining studies demonstrated low incidence of CIN, with values between 1.9% to 4.9% (Table 2). When using another reference value of 6.4%, the average rate of CIN in another meta-analysis by McDonald et al. [35] with most studies focusing on non-ED settings, three additional studies are classified as having low CIN incidence. Nevertheless, high CIN incidence was still reported in a greater number of studies (n = 12). Overall, this suggests that the CIN incidence in the ED population is relatively higher than that in the non-ED setting.

#### 3.3.2. CIN Definition in Various Studies

The most common definition was an absolute increase of ≥0.5 mg/dL or ≥25% increase over baseline sCr, occurring within 48 to 72 h (n = 4). The CIN incidences reported for the studies were 4.9% [12], 5.69% [31], 6.8% [27], and 7.5% [18]. The incidences were relatively similar despite having a different patient population in the Hong et al. [18] study compared to the other three studies. Furthermore, three other studies describing CIN using another definition of an absolute increase of ≥0.5 mg/dL or ≥25% increase over baseline sCr, within 2 to 7 days also demonstrated comparable incidences of 12% [34], 11% [32], and 14% [30]. 

However, there are studies reporting varied CIN incidences despite using the same definition. Both McGillicuddy [13] and Akman and Bakirdogen [22] used an absolute increase of ≥0.5 mg/dL or ≥25% increase over baseline sCr within 72 h but demonstrated vastly different CIN incidences of 1.9% and 36.9%, respectively. This large difference could be attributed to the dissimilarities between the two studies in terms of the sample size and the type of patients recruited. McGillicuddy [13] recruited a larger sample size of 822, focusing only on ED patients aged ≥55 years, whereas the Akman and Bakirdogen [22] study had a smaller sample size of 122 with ED patients aged ≥18 years. Moreover, this small sample size of 122 could have resulted in the large difference in CIN incidence between the Akman and Bakirdogen [22] study and the other 15 studies that also reported higher CIN incidence than the reference value of 4.96%. Additionally, the abovementioned definitions had different post-CECT sCr collection times, which could have influenced the CIN incidence.

Similarly, Hinson et al. [27] and Cho et al. [24] reported CIN incidences of 10.6% and 6.49%, respectively, using the CIN definition of an increase in sCr ≥ 0.3 mg/dL or ≥1.5 to 1.9-fold increase from baseline sCr, within 48 to 72 h. However, this difference was likely due to the difference in volume of ICM used for varying CT scan regions [36]. The Hinson et al. [27] study required a greater ICM volume ranging from 80 to 120 mL for CT scans of all body regions, whereas Cho et al. [24] used 60 mL of ICM as it focused on chest CT scans only. 

Overall, the definition used impacted the CIN incidence reported, but reasons for choosing a particular definition were not mentioned in most studies (n = 12). Only six studies justified their definition by stating that it was a common criterion used in various literatures [13,20,21,26,27,28]. Hence, the definitions will be further discussed in terms of the sCr measurements and the post-CECT sCr collection time used.

##### sCr Measurement

Eight different measurements of sCr were mentioned. An absolute increase of ≥0.5 mg/dL or ≥25% increase over baseline sCr was used in 11 out of 18 studies, with the lowest incidence at 1.9% and the highest at 36.9%. Of these, three studies investigated the incidence of CIN using an additional sCr measurement [26,27,32]. The second definition from Puchol et al. [26] defined CIN as an absolute increase of ≥0.3 mg/dL or 1.3 times greater than baseline sCr. With this definition, a higher CIN incidence at 7.72% was demonstrated compared to an incidence of 7.15% when the first definition was used. Likewise, CIN incidence was higher at 11% when CIN was defined as an absolute rise in sCr of ≥0.3 mg/dL regardless of a 25% increase in sCr compared to the 6% when the first definition was used [32]. Hinson et al. [27] also observed the same pattern where the additional definition of an increase in sCr ≥ 0.3 mg/dL or ≥1.5 to 1.9-fold increase from baseline sCr resulted in a higher incidence of CIN of 10.6%. This was 3.8% more than when the first definition was used. Compared to the first definition, the alternative criteria for all three studies were less stringent, which resulted in a higher incidence of CIN. However, this may have led to a possibility of more false positives.

An absolute sCr increase of ≥0.3 mg/dL or ≥1.5-fold increase over baseline sCr was also used to define CIN in three different studies and they reported varying incidence of 6.2% [21], 7.2% [23], and 13.2% [20]. Hence, besides factors such as sample size, CT scan region, and patient characteristics, the sCr value and range utilized in the definition of CIN is critical and could have contributed to the wide variation in CIN incidence.

##### Post-CECT sCr Collection Time 

Five different sCr collection time intervals were used, which could have contributed to the differences in CIN incidence. CIN was most often defined within 48 to 72 h (n = 8) (Table 2). Comparing between studies by Mitchell et al. [32] and Dağar et al. [12], Dağar et al. [12] found a CIN incidence of 4.9%, which was lower than the CIN incidence of 11% revealed by Mitchell et al. [32]. Although both studies used an absolute increase of ≥0.5 mg/dL or ≥25% increase over baseline sCr to define CIN, Mitchell et al. [32] allowed for a longer inclusion time frame of up to 7 days, while Dağar et al. [12] utilized the common collection time interval of 48 to 72 h. The shorter inclusion period could have contributed to the lower CIN incidence as the narrow time period might have excluded some patients with CIN since it was possible for the post-CECT sCr levels to peak after 72 h [12,28]. Conversely, the longer time frame could have allowed more time for patients to develop acute kidney injury (AKI) due to other secondary hospital-acquired causes such as sepsis, instead of ICM exposure [31]. 

#### 3.3.3. CIN-Induced Complications 

Common CIN-induced complications include adverse renal events such as dialysis, CKD, end-stage renal disease (ESRD), and renal transplantation, as well as death and increased length of stay (LOS) in hospitals.

The incidence of adverse renal events is generally low in the ED. Three studies revealed that dialysis was not required in ED patients with CIN [31,33,34], whereas low incidences of dialysis were demonstrated in six studies [12,13,20,21,27,28]. Of these, studies performed by Huang et al. [28], Brito et al. [21], and Dağar et al. [12] found low incidences of temporary haemodialysis, with no patients requiring permanent haemodialysis. Hsu et al. [25] also had no patients who required chronic dialysis, despite a relatively high incidence of emergent dialysis performed. McGillicuddy et al. [13] had two CIN patients who required permanent dialysis after being discharged, but they only made up 0.3% of patients who underwent CECT in the ED. Furthermore, new initiation of dialysis and diagnosis of ESRD at 30 days for CECT patients in the ED were rare among patients with CKD receiving ICM [20]. Two studies also concluded that ICM administration during CECT in the ED does not increase the risk of emergent dialysis [25,27], diagnosis of CKD, and renal transplantation at 6 months [23]. While Cho et al. [24] reported five ED patients with CIN after CECT receiving renal replacement therapy, it was initiated due to predisposing conditions such as multiorgan failure instead of ICM exposure. Conversely, two studies showed an association between CIN development and increased risk of severe renal failure within 45 days after CECT in the ED [30,32].

However, we have found that CIN is associated with an increased risk of mortality in the ED based on a majority of the articles. Two articles [25,30] reported high incidence of short-mortality rates in their cohort, whereas Dağar et al. [12] and Hong et al. [18] reported no and low mortality respectively. Five studies [13,20,28,30,32] noted that CIN was associated with an increased risk of death, although four studies [12,18,25,31] contraindicated this finding, whereby their studies revealed no significant differences in mortality rates between the CECT and non-CECT groups. 

Moreover, it cannot be confirmed that CIN is associated with increased LOS, as Hong et al. [18] showed no association between CIN and LOS, whereas McGillicuddy et al. [13] showed that CIN was associated with an increased LOS.

#### 3.3.4. Validity of Classical Risk Factors for CIN in ED Settings

##### Positive Findings

Congestive heart failure (CHF), acute hypotension, liver diseases, and illness severity of patients were associated with CIN development in the ED. 

Three studies demonstrated the association between CHF and CIN development [23,27,29], with only Brito et al. [21] reporting otherwise. Acute hypotension can also predispose patients to CIN [12,18,28]. Moreover, patients with liver diseases such as liver cirrhosis are at a higher risk for CIN [18,29]. Hinson et al. [23] and Puchol et al. [26] further showed that CIN was associated with patients that were more severely ill.

##### Negative Findings

Age, gender, estimated glomerular filtration rate (eGFR), diabetes, vascular disease, anaemia, and smoking habits were risk factors that had no association with CIN development in the ED.

Age was not associated with increased likelihood of CIN development in ED patients after ICM injection [18,21,29,31]. Traub et al. [29] further specified that advanced age of >75 years was not an independent predictor of CIN after CECT in the ED. However, there were only three studies that reported otherwise [12,26,27]. 

Furthermore, no association was found between gender and CIN development in the ED [18,26], even when CIN incidence was measured using two different definitions [26]. However, Akman and Bakirdogen [22] found a one-fold increased risk of CIN for older females, compared to younger males after receiving ICM in the ED. 

Additionally, initial eGFR was not associated with increased risk of CIN among ED patients with normal or near-normal renal function [18,21,31]. The same relationship was also identified by Cho et al. [24] and Hinson et al. [23,27] in patients with varying renal functions, including those with eGFR < 30 mL/min/1.73 m^2^. Similarly, Mitchell et al. [30] suggested that baseline eGFR < 60 mL/min/1.73 m^2^ may not be a sensitive predictor of CIN, as it was observed that among patients with eGFR < 60 mL/min/1.73 m^2^ the percentage of patients with CIN was 7% lower than that of those without CIN. Nevertheless, Brito et al. [21] reported that eGFR < 60 mL/min/1.73 m^2^ was a predictor of CIN. 

Although pre-existing diagnosis of CKD was associated with increased likelihood of CIN during multivariable logistic regression modelling, no significant difference in risk of CIN development was found after analysis of subgroups stratified by baseline eGFR [27]. However, Kene et al. [20] discovered that patients with CKD stage 3 have a greater risk of CIN, but not CKD stage 4 to 5 patients. 

The lack of association between diabetes and the risk of CIN development was demonstrated in three studies [21,31,33], in contrast to studies by Huang et al. [28] and Traub et al. [29]. 

Subsequently, Brito et al. [21] and Traub et al. [29] demonstrated that a history of vascular disease failed to predict CIN incidence, despite CIN being more common among patients with coronary artery disease [34]. 

Anaemia [29] and smoking habits [21] were also not risk factors for CIN.

##### Inconclusive Findings

There was inconclusive evidence to determine if sCr levels and hypertension were risk factors for CIN development after receiving ICM in the ED.

In three studies, baseline sCr was associated with a higher risk of developing CIN after receiving ICM in the ED [21,22,26]. Although Traub et al. [29] reported that pre-contrast sCr > 2.0 mg/dL is an independent predictor of CIN, Huang et al. [28] showed that a pre-contrast sCr > 1.5 mg/dL was a risk factor only in elderly patients. Conversely, three other studies observed that initial sCr cannot predict the onset of CIN after ICM administration in the ED, with no statistically significant difference in sCr levels between patients with and without CIN [18,27,31]. Furthermore, Mitchell et al. [30] found that raised pre-CECT sCr levels were not associated with a greater risk of CIN. The CIN incidence of 6% among ED patients with an elevated baseline sCr level was also lower than expected, while a higher CIN frequency of 15% was reported for those with normal baseline sCr [34]. 

It is also unclear if hypertension is a CIN predictor due to contradicting results from Traub et al. [29] and Brito et al. [21].

#### 3.3.5. ICM Administration and AKI Development

Studies comparing between CECT and non-CECT groups in the ED revealed no significant difference in AKI incidence and multivariable logistic regression analysis demonstrated no independent effect of ICM administration on the risk of AKI development [13,21,23,25,26,27]. This relationship was demonstrated across different groups of the ED patient population, which comprises AIS patients [21], elderly trauma patients [13], and patients with sepsis [23,25]. Moreover, CIN development in the ED was neither affected by the type nor volume of ICM injected [33]. However, only Kene et al. [20] reported that ICM was associated with greater risk of developing AKI. These findings suggest that the impact of ICM administration during CECT on AKI development in the ED could be less significant than other predisposing factors.

## 4. Discussion

### 4.1. Findings

To the best of our knowledge, this is the first systematic review to evaluate the incidence of CIN after CECT in the ED. Our findings suggest that the majority of the studies reported a higher CIN incidence in ED settings even when two different reference values, mostly in non-ED settings, were used for comparison. This contradicts results from other meta-analyses that reported a low incidence of CIN among CECT patients [19,37]. However, this discrepancy could be due to the inclusion of more studies with non-ED patients in their meta-analyses. Unlike the ED setting where patients require emergent CECT, there is ample time in the non-ED setting for more prophylactic treatments before CECT to reduce the risk of developing CIN, and CECT could also be postponed if the patient’s condition is not optimal. Moreover, clinicians could avoid using ICM for patients at high risk of CIN in non-emergent cases by substituting CECT with non-CECT or another imaging modality. Therefore, this results in a lower CIN incidence reported in studies with non-ED patients.

Furthermore, significant variations in CIN incidence were found across the 18 literatures included, which could be caused by differences in sCr measurement and post-CECT sCr collection time. This has also been discussed in numerous studies attributing the differences in CIN incidence to the different definitions used [12,18,19,25,38]. Likewise, Aycock et al. [39] revealed that depending on the definition, the incidence of CIN would fluctuate; however, although it was statistically significant, they doubted its clinical significance. A similar observation was also reported by Guillon et al. [40], whereby CIN incidence reported using three definitions that differed greatly from each other despite using the same group of patients after coronary angiography. Additionally, the usage of the terms “absolute” and “relative” in the CIN definition could also have influenced the incidence of CIN as these two definitions of CIN were not interchangeable [41]. Differences in CIN incidence could also be due to the different ethnic compositions present within the study populations, which were not specified. An earlier study reported that the risk of CIN after percutaneous coronary intervention differs, whereby CIN incidence was higher among African Americans than Whites, and lowest in Asians [42]. Thus, this suggests that CIN incidence may be higher in studies with a greater proportion of patients belonging to a specific ethnicity than another. Furthermore, the high incidence of CIN and mortality reported in some studies could have been due to their patient exclusion criteria whereby they failed to exclude patients with a history of diabetes mellitus, end-stage renal failure, and baseline creatinine levels below 0.4 mg/dL or greater than or equal to 4.0 mg/dL. This could have led to the review of a cohort with generally higher risk of CIN, thus leading to a higher incidence rate of CIN and mortality.

Moreover, the risk of CIN-induced complications after receiving ICM in ED settings is low. For example, there is no or low incidence of patients requiring chronic dialysis, with some studies reporting a lack of association between CECT and risk of emergent dialysis and this is corroborated by existing meta-analyses [35,37,39]. 

Overall, the increased risk of CIN development after receiving ICM in the ED was associated with CHF, liver cirrhosis, and acute hypotension, which coincides with the risk factors mentioned by Modi et al. [43] and Shams and Mayrovitz [44]. However, age, gender, eGFR, diabetes, anaemia, and vascular disease were not risk factors for developing CIN after ICM administration in the ED, which contradicts the above two studies. These imply that the onset of CIN is unpredictable as classical risk factors identified in largely non-ED settings are not necessarily applicable in the ED. 

Furthermore, our findings demonstrate the lack of significant difference in AKI incidence between CECT patients and non-CECT patients in the ED, which coincides with the results from meta-analyses by Aycock et al. [39] and Lee et al. [45] that included studies from various clinical settings. Additionally, in the ED setting, the volume of ICM injected has no association with CIN development, which concurs with findings from Moos et al. [19] and Kooiman et al. [37]. These suggest that, compared to ICM administration, other predisposing factors such as patients’ conditions are likely to have a more significant contribution to AKI development in the ED [12,29,46]. This is also in line with recent consensus statements indicating that the risk of AKI development after intravenous administration of ICM has been overstated for patients with decreased renal function [5,47,48,49].

### 4.2. Limitations

This systematic review has several limitations. Firstly, the studies included were largely retrospective and observational in nature, which resulted in an inherent selection bias. Since data were usually retrieved from the hospital database, there may be a lack of pertinent details required to control for all confounding variables. While six out of eighteen studies attempted to control for potential confounders using propensity score matching by alleviating systematic differences between baseline characteristics among their subjects, its effects were limited due to the inability to account for unmeasured confounders unlike randomization [50]. Moreover, a common requirement in all retrospective studies was to have a post-CECT sCr measurement, which might have been unavailable for discharged patients. Thus, most ED patients included in the studies could be those requiring hospital admission after CECT. This group of admitted patients might have been more ill and could possibly have been at a greater risk for CIN development compared to those who were discharged, hence introducing more selection bias. Furthermore, creatinine levels in these patients are known to have substantial daily fluctuations with or without the administration of CM, which influences the reliability of the CIN incidence reported [51,52]. There is also the possibility of data being inaccurately recorded in the database, which cannot be controlled for and may cause measurement bias. 

Secondly, in both prospective and retrospective studies, the care of patients and the decision for undergoing CECT would be influenced by various factors, such as the patient’s condition, the local clinical practice and the clinician’s preference. This results in selection bias as patients in the CECT group for each study may potentially be at a higher or lower risk for CIN. Hence, the CIN incidence reported by the studies may be under- or overestimated. While selection bias can be minimized using randomized controlled trials (RCTs) [53], it would be unethical to randomly assign patients into the CECT group and disregard the patient’s health and clinical indications. Thus, no such RCTs have been conducted. 

Thirdly, all 18 studies attempted to address potential confounders such as excluding patients who received intravenous ICM within 7 days before the ED visit. Nonetheless, the extent to which confounders were controlled for differed across the studies. Furthermore, prophylactic treatments before CECT, a potential confounder, were used in some studies. These factors could reduce the incidence of CIN [54,55] and thus impact the reliability of reported CIN incidence. However, recent studies have failed to demonstrate the efficacy of prophylaxis in decreasing the risk of CIN and dialysis after ICM exposure, which makes the impact of this confounder less significant [56,57].

Additionally, there is significant heterogeneity across the 18 studies, such as the type of patients and volume of ICM used, due to the inclusion of literatures involving CECT in the ED with age being the only restriction. The variability in study characteristics may make it difficult to interpret and compare the results across the studies. However, conclusions about CIN after CECT in ED settings could be more generalizable to the adult population as a result.

Although we restricted our eligibility criteria to only English papers, no literature was excluded because of the language used. However, publication bias may still be present as methods besides database searching, such as handsearching, were not utilized to identify other relevant literature. Another potential limitation of this study is that this study has not been registered to PROSPERO.

## 5. Conclusions

In conclusion, the majority of studies demonstrated a higher CIN incidence following ICM administration in the ED when compared to the reference values. We found no consensus on the definition of CIN in many published studies, with varying sCr measurements and post-CECT sCr collection times. These could have contributed to the wide variation in reported CIN incidence. Nonetheless, the risk of CIN-induced complications after ICM injection in ED settings such as long-term dialysis is very low. Despite the higher CIN incidence reported, the impact of ICM administration on AKI development in the ED is likely less significant compared to other predisposing factors. Moreover, not all classical CIN risk factors, such as age and eGFR, remain valid in the ED to predict CIN onset. Nevertheless, ICM administration during CECT for ED adult patients should still be clinically justified with the benefits outweighing its risks because the CIN-induced mortality in the ED setting is relatively high.

## Figures and Tables

**Table 1:** Full search strategy.

Databases	Search Terms		Results
PubMed	#1	“contrast-induced nephropathy”[tw] OR “contrast induced nephropathy”[tw] OR “CIN”[tw] OR “renal disorder”[tw] OR “nephrosis”[tw] OR “nephropathy”[tw] OR “nephrotoxicity”[tw] OR “acute kidney injury”[tw] OR “AKI”[tw] OR “kidney disease”[tw] OR “Acute Kidney Injury”[Mesh] OR “Kidney Diseases/chemically induced”[Mesh]	236,637
	#2	“Contrast enhanced Computed tomography”[tw] OR “CECT”[tw] OR “CT”[tw] OR “computed tomography”[tw] OR “CT angio*”[tw] OR “CTA”[tw] OR “Tomography, X-ray Computed” [Mesh]	745,818
	#3	“ED”[tw] OR “ER”[tw] OR “trauma”[tw] OR “emergency”[tw] OR “Emergency Service, Hospital”[Mesh]	725,803
	#4	#1 AND #2 AND #3	365
Cochrane	#1	MeSH descriptor: [Acute Kidney Injury] explode all trees	1540
	#2	MeSH descriptor: [Kidney Diseases] explode all trees and with qualifier(s): [chemically induced—CI]	840
	#3	(“contrast-induced nephropathy”):ti,ab,kw OR (“contrast induced nephropathy”):ti,ab,kw OR (“CIN”):ti,ab,kw OR (“renal disorder”):ti,ab,kw OR (“nephrosis”):ti,ab,kw	2130
	#4	(“nephropathy”):ti,ab,kw OR (“nephrotoxicity”):ti,ab,kw OR (“acute kidney injury”):ti,ab,kw OR (“AKI”):ti,ab,kw OR (“kidney disease”):ti,ab,kw	26,657
	#5	#1 OR #2 OR #3 OR #4	27,810
	#6	MeSH descriptor: [Tomography, X-Ray Computed] explode all trees	5206
	#7	(“Contrast enhanced Computed tomography”):ti,ab,kw OR (“CECT”):ti,ab,kw OR (“CT”):ti,ab,kw OR (“computed tomography”):ti,ab,kw OR (“CTA “):ti,ab,kw	86,780
	#8	(“CT angiogram”):ti,ab,kw OR (“CT angiography”):ti,ab,kw	1003
	#9	#6 OR #7 OR #8	87,767
	#10	MeSH descriptor: [Emergency Service, Hospital] explode all trees	2550
	#11	(“ED”):ti,ab,kw OR (“ER”):ti,ab,kw OR (“trauma”):ti,ab,kw OR (“emergency”):ti,ab,kw	84,580
	#12	#10 OR #11	84,580
	#13	#5 AND #9 AND #12	115
Web of Science	#1	TS = (“contrast-induced nephropathy” OR “contrast induced nephropathy” OR “CIN” OR “renal disorder” OR “nephrosis” OR “nephropathy” OR “nephrotoxicity” OR “acute kidney injury” OR “AKI” OR “kidney disease”)	237,874
	#2	TS = (“Contrast enhanced Computed tomography” OR “CECT” OR “CT” OR “computed tomography” OR “CT angiogra*” OR “CTA”)	640,682
	#3	TS = (“ED” OR “ER” OR “trauma” OR “emergency”)	777,572
	#4	#1 AND #2 AND #3	312

## Data Availability

Not applicable.

## Appendix A

**Table A1:** Complete Synthesis of Themes.

Article	Codes	Summary	Subthemes/ Themes	Themes
(Kene et al., 2021) [20]	High incidence of CIN: 13.2%	With reference to the pooled CIN incidence of 4.96% reported in the meta-analysis by Moos et al. (2013), values **above** 4.96% were categorized as **high** incidence of CIN.	Overall CIN Incidence	
(Hinson et al., 2019) [23]	High incidence of CIN: 7.2%			
(Mitchell & Kline, 2007) [34]	High incidence of CIN: 12%			
(Brito et al., 2020) [21]	High incidence of CIN: 6.2%			
(Hong et al., 2016) [18]	High incidence of CIN: 7.5%			
(Sinert et al., 2012) [31]	High incidence of CIN: 5.69%			
(Mitchell et al., 2010 [32]	High incidence of CIN: 11% and 6%			
(Cho et al., 2019) [24]	High incidence of CIN: 6.49%			
(Mitchell et al., 2012) [30]	High incidence of CIN: 14%			
(Huang et al., 2013) [28]	High incidence of CIN: 8.6%			
(Traub et al., 2013) [29]	High incidence of CIN: 7%			
(Hinson et al., 2017) [27]	High incidence of CIN: 6.8% and 10.6%			
(Hsu et al., 2019) [25]	High incidence of CIN: 12.4%			
(Puchol et al., 2019) [26]	High incidence of CIN: 7.15% and 7.72%			
(Akman & Bakirdogen, 2020) [22]	High incidence of CIN: 36.9%			
(McGillicuddy et al., 2010) [13]	Low incidence of CIN: 1.9%	With reference to the pooled CIN incidence of 4.96% reported in the meta-analysis by Moos et al. (2013), values **below** 4.96% were categorized as **low** incidence of CIN.		
(Hopyan et al., 2008) [33]	Low incidence of CIN: 2.9%			
(Dağar et al., 2020) [12]	Low incidence of CIN: 4.9%			
(Kene et al., 2021 [20]; Brito et al., 2020 [21]; Hinson et al., 2019 [23])	Absolute sCr increase of ≥0.3 mg/dL or ≥1.5-fold increase over baseline sCr	The different serum creatinine (sCr) measurements used in the definition of nephropathy	sCr Measurement	CIN Definitions in various studies
(Akman & Bakirdogen, 2020 [22]; Dağar et al., 2020 [12]; Puchol et al., 2019 [26]; Hinson et al., 2017 [27]; Hong et al., 2016 [18]; Traub et al., 2013 [29]; Mitchell et al., 2012 [30]; Sinert et al., 2012 [31]; McGillicuddy et al., 2010 [13]; Mitchell et al., 2010 [32]; Mitchell & Kline, 2007 [34])	Absolute increase of ≥0.5 mg/dL or ≥25% increase over baseline sCr			
(Mitchell et al., 2010) [32]	An absolute rise in sCr of ≥0.3 mg/dL			
(Cho et al., 2019 [24]; Hinson et al., 2017 [27])	Increase in sCr ≥0.3 mg/dL or ≥1.5 to 1.9-fold increase from baseline sCr			
(Hopyan et al., 2008) [33]	≥25% increase in baseline sCr			
(Huang et al., 2013) [28]	Increase in sCr ≥ 0.5 mg/dL			
(Hsu et al., 2019) [25]	Absolute increase of 0.5 mg/dL or >50% increase in baseline sCr			
(Puchol et al., 2019) [26]	Absolute increase of ≥0.3 mg/dL or 1.3 times greater than baseline sCr			
(Kene et al., 2021 [20]; Puchol et al., 2019 [26])	24 to 72 h	The different timings of sCr follow-ups after CECT used in the definition of nephropathy	Post-CECT sCr Collection Time	
(Dağar et al., 2020 [12]; Cho et al., 2019 [24]; Hinson et al., 2019 [23]; Hsu et al., 2019 [25]; Hinson et al., 2017 [27]; Hong et al., 2016 [18]; Huang et al., 2013 [28]; Sinert et al., 2012 [31])	48 to 72 qh			
(Akman & Bakirdogen, 2020 [22]; Brito et al., 2020 [21]; McGillicuddy et al., 2010 [13]; Hopyan et al., 2008 [33])	Within 72 h			
(Mitchell et al., 2012, [30], 2010 [32]; Mitchell & Kline, 2007 [34])	2 to 7 days			
(Traub et al., 2013) [29]	48 to 96 h			
(Sinert et al., 2012 [31]; Hopyan et al., 2008 [33]; Mitchell and Kline, 2007 [34])	No dialysis was required	Complications of CIN include adverse renal events such as dialysis, chronic kidney disease, end-stage renal disease, and renal transplantation.	CIN-induced Complications	
(Kene et al., 2021 [31]; Brito et al., 2020 [21]; Dağar et al., 2020 [12]; Huang et al., 2013 [28]; McGillicuddy et al., 2010 [13]; Hinson et al., 2017 [27])	Low incidence of dialysis			
(Hsu et al., 2019) [25]	High incidence of dialysis			
(Brito et al., 2020 [21]; Dağar et al., 2020 [12]; Hsu et al., 2019 [25]; Huang et al., 2013 [28])	Temporary haemodialysis only, none required permanent dialysis			
(McGillicuddy et al., 2010) [13]	Require permanent dialysis			
(Cho et al., 2019) [24]	Renal replacement therapy required for 5 patients			
(Hsu et al., 2019 [25]; Hinson et al., 2017 [27])	IV administration of contrast does not increase the risk of emergent dialysis			
(Hinson et al., 2019) [23]	IV administration of contrast does not increase the risk of diagnosis of CKD and renal transplantation at 6 months			
(Mitchell et al., 2010 [32], 2012 [30])	Association between CIN development and higher risk of severe renal failure within 45 days			
(Kene et al., 2021 [20]; Hsu et al., 2019 [25]; Huang et al., 2013 [28]; Mitchel et al., 2012 [30]; McGillicuddy et al., 2010 [13])	CIN is associated with an increased risk of death	Mortality is another complication of CIN		
(Hsu et al., 2019 [25]; Hong et al., 2016 [18]; Sinert et al., 2012 [31])	No significant differences in mortality rates between the CECT and non-CECT groups			
(Hong et al., 2016) [18]	No association between CIN and LOS	Increased length of stay (LOS) is also a complication of CIN		
(McGillicuddy et al., 2010) [13]	CIN was associated with an increased LOS			
(Hinson et al., 2019 [23]; Hinson et al., 2017 [27]; Traub et al., 2013 [29])	Congestive heart failure was associated with development of CIN	Congestive heart failure, acute hypotension, liver diseases, and illness severity of patients are associated with development of CIN in the ED.	Positive Findings	Validity of Classical Risk factors for CIN in ED settings
(Brito et al., 2020) [21]	Congestive heart failure was not a predictor of CIN			
(Dağar et al., 2020 [12]; Hong et al., 2016 [18]; Huang et al., 2013 [28])	Patients with acute hypotension are at a higher risk for CIN			
(Hong et al., 2016 [18]; Traub et al., 2013 [29])	Patients with liver diseases such as liver cirrhosis are at a higher risk of CIN			
(Hinson et al., 2019 [23]; Puchol et al., 2019 [26])	CIN was associated with patients that were more severely ill			
(Dağar et al., 2020 [12]; Puchol et al., 2019 [26]; Hinson et al., 2017 [27])	Age is associated with an increased likelihood of CIN development	Age, gender, eGFR, diabetes, vascular disease, anaemia, and smoking habits were not associated with CIN development in the ED.	Negative Findings	
(Brito et al., 2020 [21]; Hong et al., 2016 [18]; Traub et al., 2013 [29]; Sinert et al., 2012 [31])	Age is not associated with risk of developing CIN			
(Puchol et al., 2019 [26]; Hong et al., 2016 [18])	Gender is not associated with CIN development			
(Akman & Bakirdogen, 2020) [22]	Older females associated with higher risk of CIN, compared to males who are younger			
(Brito et al., 2020 [21]; Cho et al., 2019 [24]; Hinson et al., 2019 [23]; Hinson et al., 2017 [27]; Hong et al., 2016 [18]; Sinert et al., 2012 [31])	No association between eGFR and CIN			
(Mitchell et al., 2012) [30]	eGFR < 60 mL/min/1.73 m^2^ may be an insensitive predictor of CIN after CTPA			
(Brito et al., 2020) [21]	eGFR < 60 mL/min/1.73 m^2^ is a predictor of CIN			
(Kene et al., 2021) [20]	Patients with CKD stage 3 at higher risk of AKI, but not for CKD 4–5 patients			
(Hinson et al., 2017) [27]	Pre-existing diagnosis of CKD was associated with increased likelihood of CIN by multivariable logistic regression modelling			
(Brito et al., 2020 [21]; Sinert et al., 2012 [31] Hopyan et al., 2008 [33])	No association between CIN and diabetes			
(Huang et al., 2013 [28]; Traub et al., 2013 [29])	Diabetes mellitus was a risk factor for CIN			
(Mitchell & Kline, 2007) [34]	A relatively high AKI frequency among those with coronary artery disease			
(Brito et al., 2020 [21]; Traub et al., 2013 [29])	History of vascular disease failed to predict CIN			
(Traub et al., 2013) [29]	Anaemia was not a risk factor of CIN			
(Brito et al., 2020) [21]	Smoking habits was not a predictor of CIN.			
(Brito et al., 2020 [21]; Akman & Bakirdogen, 2020 [22]; Puchol et al., 2019 [26])	Baseline sCr was associated with a higher risk of developing CIN	There was inconclusive evidence to conclude if sCr levels and hypertension were risk factors for CIN development after receiving iodinated contrast in the ED.	Inconclusive Findings	
(Huang et al., 2013) [28]	Pre-contrast sCr of more than 1.5 mg/dL was a risk factor for CIN			
(Traub et al., 2013) [29]	Pre-contrast creatinine level > 2.0 mg/dL is an independent predictor of CIN, but not for creatinine > 1.5 mg/dL			
(Hinson et al., 2017 [27]; Hong et al., 2016 [18]; Sinert et al., 2012 [31])	sCr level is not associated with risk of CIN			
(Mitchell et al., 2012) [30]	Elevated sCr measurement was not associated with an increased risk of CIN following CTPA			
(Mitchell & Kline, 2007) [34]	Laboratory-defined CIN occurred at a lower than expected frequency among those with an elevated baseline sCr concentration (6% vs. 15% among those with normal baseline sCr).			
(Traub et al., 2013) [29]	Hypertension was a predictor of CIN			
(Brito et al., 2020) [21]	Hypertension is not a predictor of AKI			
(Kene et al., 2021) [20]	Administration of iodinated contrast is associated with increased risk of CIN development	The relationship between iodinated contrast media (ICM) and development of acute kidney injury (AKI).	ICM Administration and AKI Development	
(Brito et al., 2020 [21]; Hinson et al., 2019 [23]; Puchol et al., 2019 [26]; Hsu et al., 2019 [25]; Hinson et al., 2017 [27]; McGillicuddy et al., 2010 [13]; Hopyan et al., 2008 [33])	No association between administration of iodinated contrast and development of CIN			
